# Coupling of LETM1 up-regulation with oxidative phosphorylation and platelet-derived growth factor receptor signaling via YAP1 transactivation

**DOI:** 10.18632/oncotarget.11456

**Published:** 2016-08-20

**Authors:** Jandee Lee, Woo Kyung Lee, Mi-Youn Seol, Seul Gi Lee, Daham Kim, Hyunji Kim, Jongsun Park, Sang Geun Jung, Woong Youn Chung, Eun Jig Lee, Young Suk Jo

**Affiliations:** ^1^ Department of Surgery, Open NBI Convergence Technology Research Laboratory, Severance Hospital, Yonsei Cancer Center, Yonsei University College of Medicine, Seoul, Korea; ^2^ Department of Internal Medicine, Open NBI Convergence Technology Research Laboratory, Severance Hospital, Yonsei Cancer Center, Yonsei University College of Medicine, Seoul, Korea; ^3^ Department of Pharmacology, Metabolic Diseases and Cell Signaling Laboratory, Research Institute for Medical Sciences, College of Medicine, Chungnam National University, Daejeon, Korea; ^4^ Department of Gynecological Oncology, Bundang CHA Medical Center, CHA University, Gyeonggi-do, Korea

**Keywords:** LETM1, metabolism, prognosis, electron transport chain, cell proliferation

## Abstract

Persistent cellular proliferation and metabolic reprogramming are essential processes in carcinogenesis. Here, we performed Gene Set Enrichment Analysis (GSEA) and found that that LETM1, a mitochondrial calcium transporter, is associated with cellular growth signals such as platelet-derived growth factor (PDGF) receptor signaling and insulin signaling pathways. These results were then verified by qRT-PCR and immnunoblotting. Mechanistically, up-regulation of LETM1 induced YAP1 nuclear accumulation, increasing the expression of PDGFB, PDGFRB and THBS4. Consistent with this, LETM1 silencing caused loss of YAP1 nuclear signal, decreasing the expression of PDGFB, PDGFRB and THBS4. Immunohistochemical staining consistently indicated a positive association between LETM1 up-regulation, YAP1 nuclear localization and high PDGFB expression. In clinical data analysis, LETM1 up-regulation in thyroid cancer was found to be related to aggressive tumor features such as lymphovascular invasion (LVI, *P* < 0.001) and lymph node metastasis (LNM, *P* = 0.011). Multivariate analysis demonstrated that LETM1 up-regulation increases the risk of LVI and LNM (OR = 3.455, 95% CI = 1.537–7.766 and OR = 3.043, 95% CI = 1.282–7.225, respectively). Collectively, these data suggest that up-regulation of LETM1 induces sustained activation of proliferative signaling pathways, such as PDGF signal pathway by AKT induced YAP1 transactivation, resulting in aggressive thyroid cancer phenotypes.

## INTRODUCTION

Most cases of thyroid cancer, the most common endocrine malignancy, have a generally favorable prognosis [1]. However, 10–15% of thyroid cancers present as persistent or recurrent disease, sometimes resulting in terminal disease [2, 3]. To develop molecular markers able to predict these persistent or recurrent cases, research over the last decade based on molecular and systems biology has remarkably improved the mechanistic understanding of thyroid carcinogenesis [4, 5]. As a result of our increased understanding of the pathogenesis of thyroid cancer, aberrant activation of MEK/ERK and/or phosphatidylinositol-3 kinase (PI3K)/AKT pathways are known to play fundamental roles in thyroid cancer progression [6, 7].

In addition to sustaining proliferative signaling, metabolic alterations driven by oncogenic signaling pathways are now understood to contribute to the development of thyroid carcinogenesis [8]. Aerobic glycolysis, also called the “Warburg effect”, drives metabolic remodeling in PTEN-deficient thyroid cells [9]. Crosstalk between oncogenic BRAFV600E kinase and the mitochondria also increases glycolytic activity in thyroid cancer cells [10]. However, our recent observation indicated that a subset of papillary thyroid cancer (PTC) is able to sustain mitochondrial capacity [11]. In agreement with this finding, human melanomas exhibit increased mitochondrial capacity and resistance to oxidative stress through the activation of melanocyte lineage-specification transcription factor (MITF) and PGC1α (PPARGC1A) [12, 13]. Furthermore, addiction to oxidative phosphorylation (OxPhos) generates drug resistance against PLX4720, a BRAF inhibitor, in BRAF-mutated melanomas [14].

Leucine zipper EF-hand containing transmembrane protein 1 (LETM1), which was first discovered in patients with Wolf-Hirschhorn syndrome (WHS), has been reported to enhance mitochondrial Ca^2+^ transport and cell proliferation [15–17]. Silencing of LETM1 expression influenced autophagy activity and provoked AMPK activation and cell cycle arrest [17]. Furthermore, LETM1 promotes AKT/PKB activation by inhibition of C-terminal modulator protein (CTMP) [18]. Supporting the proto-oncogenic properties of LETM1, human multiple tissues arrays have also demonstrated increased expression of LETM1 in various cancers, including breast, head and neck cancers [19, 20].

In this study, we found that up-regulation of LETM1 expression is related to platelet-derived growth factor (PDGF) signaling, supporting the role of LETM1 in cell proliferation and energy metabolism in PTC. Remarkably, LETM1 over-expression is also linked to nuclear retention of Yes-associated protein 1 (YAP1), which upregulates PDGFB at the transcriptional level, while LETM1 silencing has the opposite effect. Based on these findings, up-regulation of LETM1 appears to play a critical role in altering mitochondrial metabolism and tumor behavior in PTC carcinogenesis through YAP1 transactivation.

## RESULTS

### Up-regulation of LETM1 linked to PDGF signal pathway

**Figure 1:**
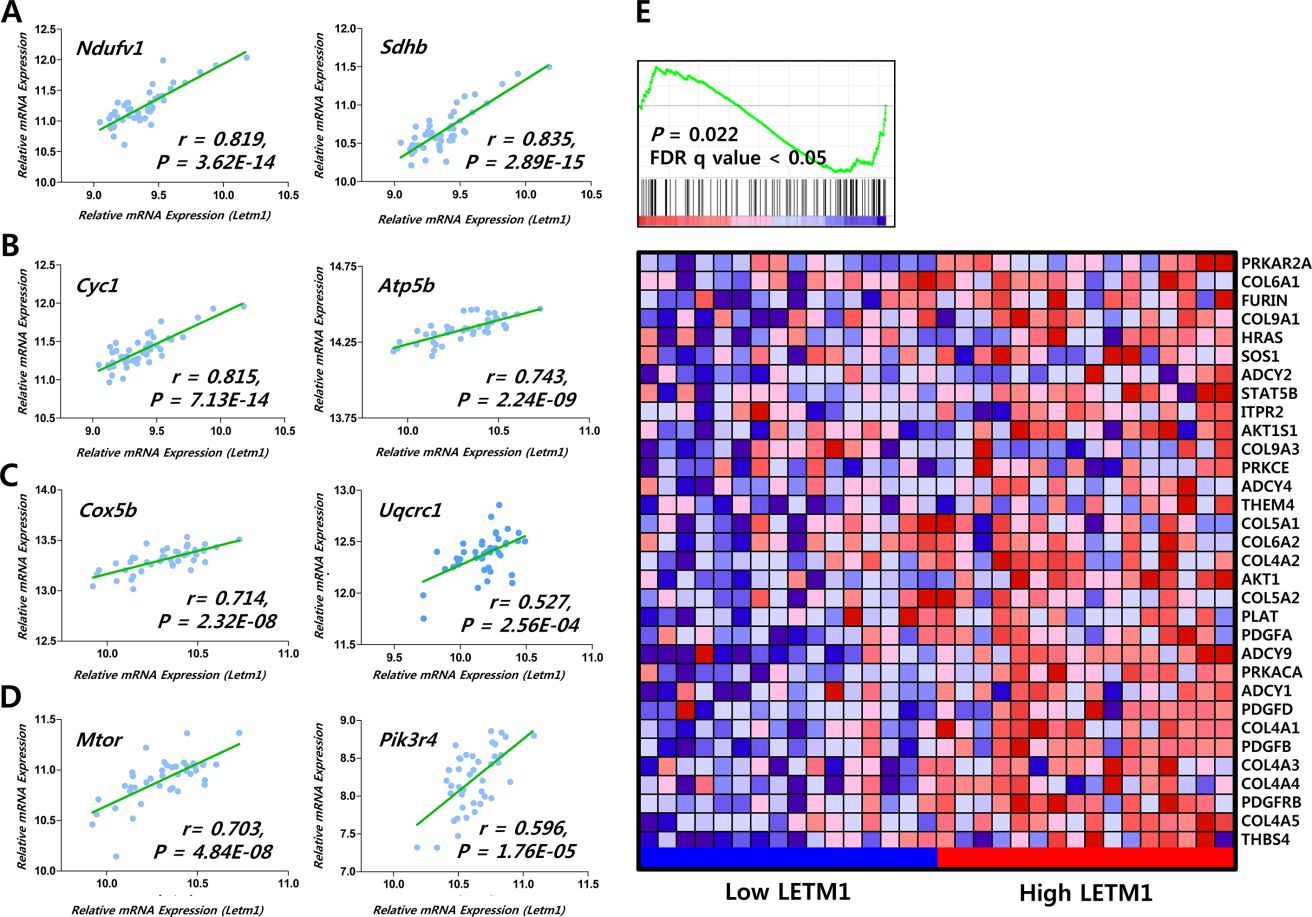
Correlation of LETM1 expression with genes related to OxPhos and cellular proliferation signaling (**A**–**D**) Correlation of *Letm1* mRNA expression with genes related to OxPhos and mTOR/PI3K signaling from public repository data in GeneNetwork (a free scientific web resource, http://www.genenetwork.org/; INIA Adrenal Affy MoGene 1.0ST (Jun12) RMA Males, EPFL/LISP BXD CD Muscle Affy Mouse Gene 1.0 ST (Nov12) RMA Exon Level). (**E**) Correlation of *LETM1* mRNA expression with genes related to the PDGF signaling pathway from the Gene Expression Omnibus (GEO) of NCBI (Gene expression data available at www.ncbi.nlm.nih.gov/projects/geo; accession no. GSE33630).

**Figure 2:**
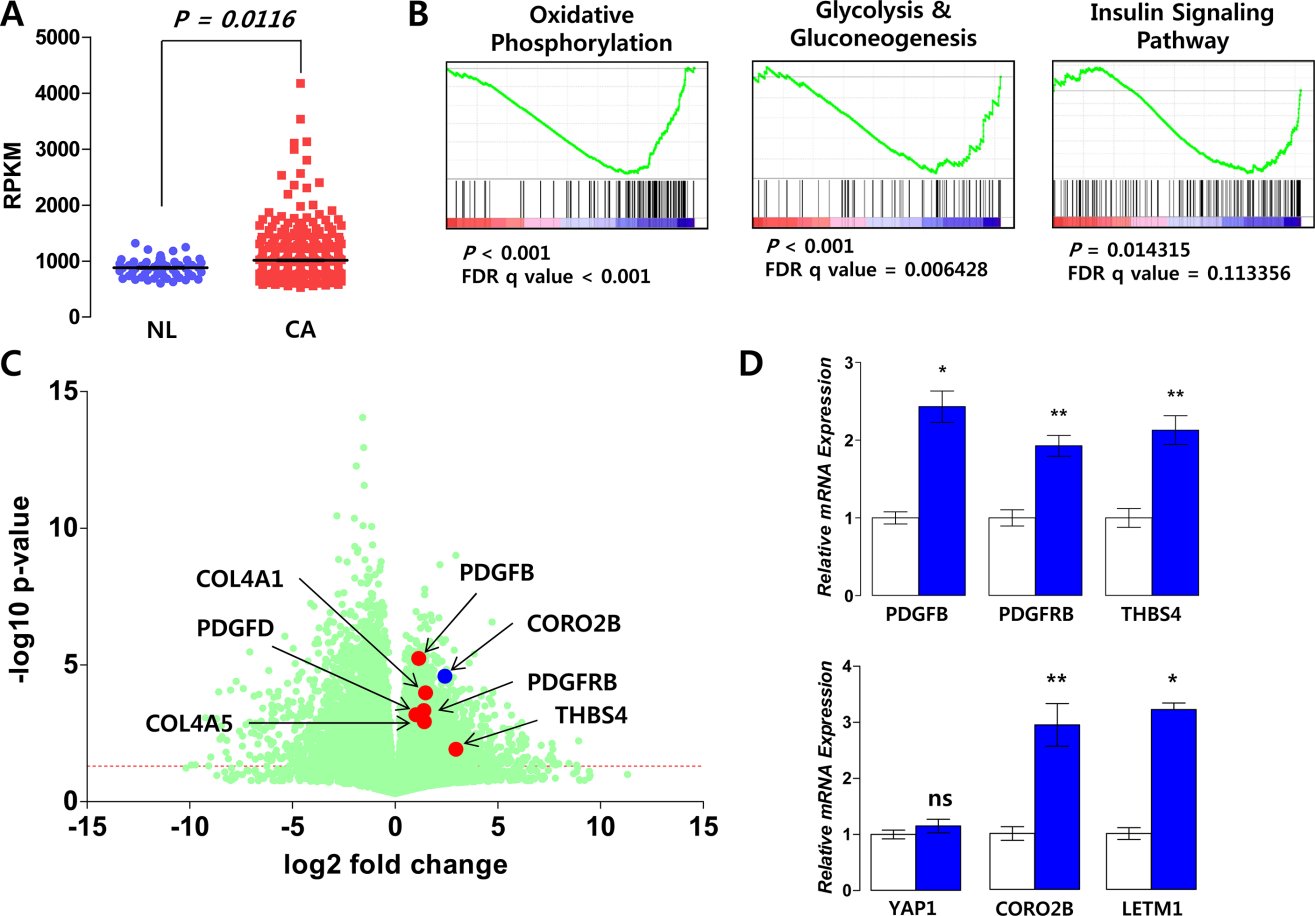
Correlation of LETM1 expression with genes related to OxPhos and cellular proliferation signaling (**A**) Comparison of *LETM1* RPKM values between normal thyroid tissues (NL) and papillary thyroid cancer (CA) using public data from The Cancer Genome Atlas Research Network (TCGA, http://cancergenome.nih.gov/). (**B**) GSEA using PTCs with the highest and lowest *LETM1* expression levels (each group *n* = 15). (**C**) Volcano plot analysis indicating increased RPKM values (log2 fold change) of central components of the PDGF signaling pathway. (**D**) qRT-PCR using validation cDNA sets from the study subjects. White and blue bars indicate matched normal thyroid tissues and PTC, respectively (each group *n* = 7). Comparisons of the average means were performed with the Mann-Whitney *U-test*. Data are presented as the means ± SD. ^**^ *P* < 0.01, ^***^ *P* < 0.001. All *P*-values are two-sided.

First, to verify our hypothesis that LETM1 expression might be linked to the cell growth signaling pathway, we performed correlation analysis using public data (EPFL/LISP BXD CD Muscle Affy Mouse Gene 1.0 ST (Nov12) RMA Exon Level, muscle mRNA; number of samples = 42) deposited at GeneNetwork. Compatible with previous reports, *Letm1* showed positive correlations with *Ndufv1* (encoding NADH:Ubiquinone Oxidoreductase Core Subunit V1), *Sdhb* (Succinate Dehydrogenase Complex Iron Sulfur Subunit B) (Figure 1A), *Cyc1* (Cytochrome C1), *Atp5b* (ATP Synthase, H^+^ Transporting, Mitochondrial F1 Complex, Beta Polypeptide) (Figure 1B), *Mrpl12* (Mitochondrial Ribosomal Protein L12) (INIA Adrenal Affy MoGene 1.0ST (Jun12) RMA Males, adrenal mRNA; number of samples = 46, Supplementary Figure S1A), *Ndufb7* (NADH:Ubiquinone Oxidoreductase Subunit B7), *Cox5b* (Cytochrome C Oxidase Subunit 5B), *Uqcrc1* (Ubiquinol-Cytochrome C Reductase Core Protein I), *Mrpl39* (Mitochondrial Ribosomal Protein L39) and *Tomm40* (Translocase Of Outer Mitochondrial Membrane 40) (EPFL/LISP BXD CD Muscle Affy Mouse Gene 1.0 ST (Nov12) RMA Exon Level) (Figure 1C, Supplementary Figure S1B–S1D). Interestingly, *Letm1* expression positively correlated with mechanistic target of rapamycin (*Mtor*) and phosphoinositide-3-kinase, regulatory subunit 4 (*Pik3r4*) (Figure 1D). In addition, Gene Set Enrichment Analysis (GSEA) using public repository data (GSE33630, expression profiling by array of 11 anaplastic thyroid carcinomas (ATC), 49 PTC and 45 normal thyroids (N)) showed that platelet-derived growth factor (PDGF) signaling pathway was coordinately enriched in PTC with high LETM1 expression (*P* = 0.022, FDR q-value < 0.05, Figure 1E). To support our findings from GeneNetwork and GSE33630, we performed GSEA using public repository data from “The Cancer Genome Atlas”. The comparison of mean RPKM (Reads Per Kilobase per Million mapped reads) between normal and tumor mRNAs indicated statistically significant increase of LETM1 expression in PTC (Figure 2A). In the TCGA analysis, 15 cases out of 505 PTC (total THCA) showed high expression of LETM1. Although 15 was a small proportion of the total PTCs in THCA, only a small subset of PTCs had demonstrated aggressive behavior in the clinical setting, therefore we decided to select 30 cases; 15 cases with the highest expression (Highest LETM1 PTC) and 15 cases with the lowest expression (Lowest LETM1 PTC) of LETM1. Performing GSEA based on TPM (Transcripts per Million), gene sets related to oxidative phosphorylation, glycolysis and gluconeogenesis and insulin signaling pathway were coordinately enriched in high LETM1 group (Supplementary Table S1, Figure 2B). Remarkably, volcano plot generated from selected 30 TCGA cases used in our GSEA indicated significantly increased fold changes of representative genes of PDGF signaling pathway such as PDGFB, platelet-derived growth factor receptor, beta polypeptide (PDGFRB), Thrombospondin-4 (THBS4), collagen, type IV, alpha 1 (COL4A1), platelet derived growth factor D (PDGFD) and collagen, type IV, alpha 5 (COL4A5) (Figure 2C). qRT-PCR experiments using our own cDNA samples from patients with PTC supported our GSEA analysis. PDGFB, PDGFRB and THBS4 were increased in PTC with high LETM1 expression (high LETM1 PTC, Figure 2D, Supplementary Table S2). Many hormones or hormone-binding proteins such as PDGFB are enhanced by YAP1 overexpression in MCF10A cells. Although mRNA of YAP1 was not increased, the mRNA expression of coronin 2B (CORO2B), a representative YAP1 target gene, was significantly increased in high LETM1 PTC (Figure 2C and 2D) [21].

### Effects of LETM1 on growth factor signaling and OxPhos

**Figure 3:**
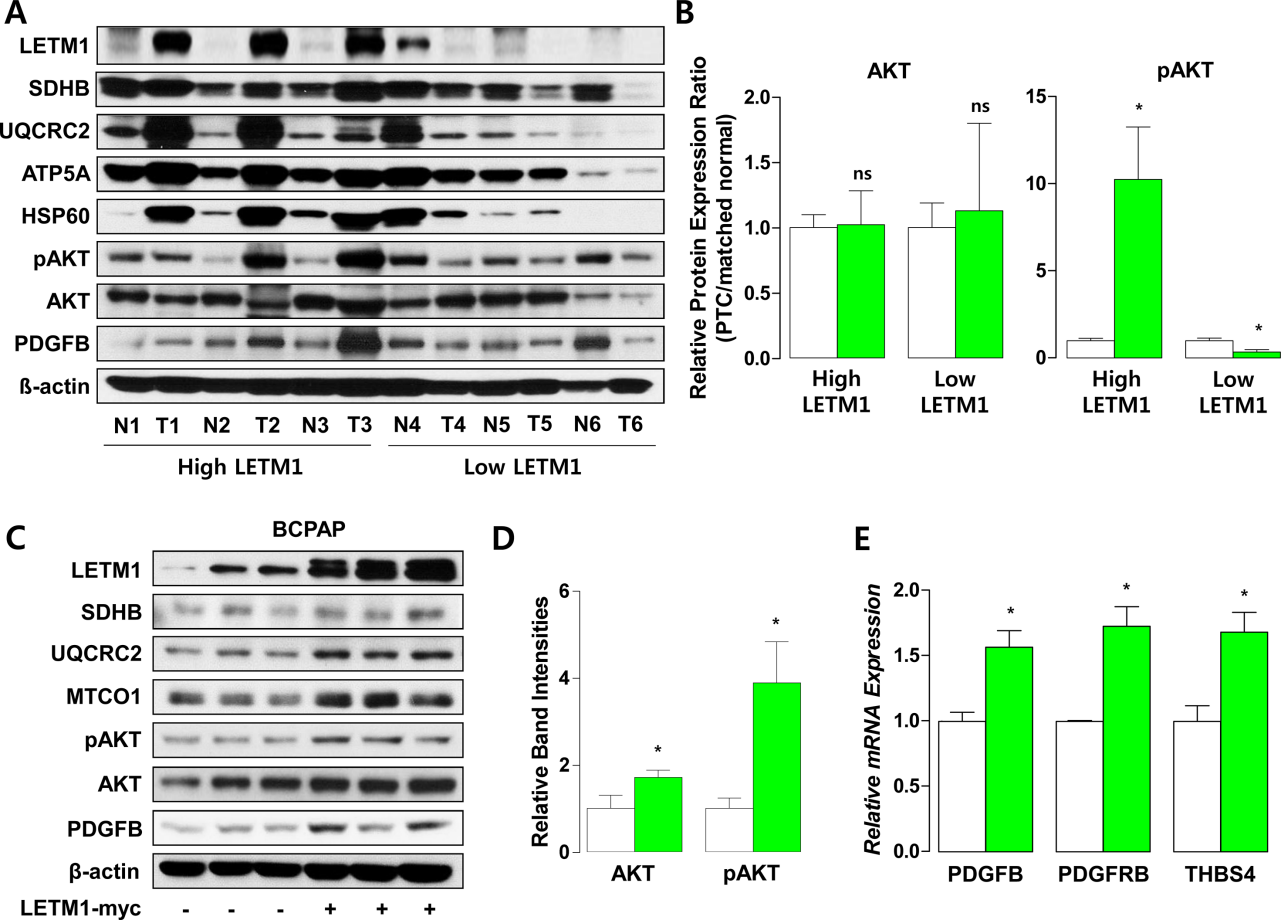
Correlation of LETM1 expression with oxidative phosphorylation (OxPhos) and cellular proliferation signaling (**A** and **B**) Representative western blot analyses indicating the association between LETM1 and OxPhos/PDGFB in tissue samples from human papillary thyroid cancers and paired normal thyroid (A), and quantification of total and phospho-AKT band intensities. White and green bars indicate matched normal thyroid tissues and PTC, respectively (B). (**C** to **E**) Representative western blot analyses indicating the effect of LETM1 overexpression on OxPhos/PDGFB in BCPAP cells (C) and quantification of total and phospho-AKT band intensities (D). qPCR analyses showing the effect of transient overexpression of LETM1 on mRNA expression of PDGFB, PDGFRB and THBS4 in BCPAP cells (E). White and green bars indicate BCPAP transfected with pcDNA3.1 and pcDNA3.1/LETM1-Myc plasmids, respectively. Comparisons of the average means were performed with the Mann-Whitney *U-test*. Data are presented as the means ± SD. ^**^P < 0.01, ^***^ *P* < 0.001. All *P*-values are two-sided.

To confirm the changes in expression of mRNAs encoding for regulators of growth factor signaling and OxPhos, we performed western blot analysis using samples derived from human thyroid cancer tissues. As shown in Figure 3A, we observed that PTC showing high LETM1 expression (high LETM1 PTC) also had high expression of components of OxPhos, such as SDHB, UQCRC2, ATP5A and HSP60, a mitochondrial heat shock protein. With regard to growth factor signaling intermediates, AKT phosphorylation (pAKT) and PDGFB were markedly increased in high LETM1 PTC, whereas pAKT and PDGFB were decreased in PTC with low LETM1 expression (low LETM1 PTC), compared to paired normal thyroid tissue (Figure 3A and 3B). When we overexpressed LETM1 in a human PTC cell line (BCPAP), we observed a coordinate increase in protein levels of OxPhos components (SDHB, UQCRC2 and MTCO1; Figure 3C). Interestingly, LETM1 overexpressing BCPAP cells showed increased glycolysis and oxygen consumption, implying that these cells uptake glucose rapidly and generate large amounts of ATP by OxPhos (Supplementary Figure S2). Furthermore, supporting the relationship between LETM1 with growth factor signaling, LETM1 overexpression increased AKT and pAKT in BCPAP (Figure 3C and 3D), associated with elevated protein levels of PDGFB and mRNA expression of PDGFB, PDGFBR and THBS4 (Figure 3C and 3E).

### Up-regulation or silencing of LETM1 is linked to YAP1 localization

**Figure 4:**
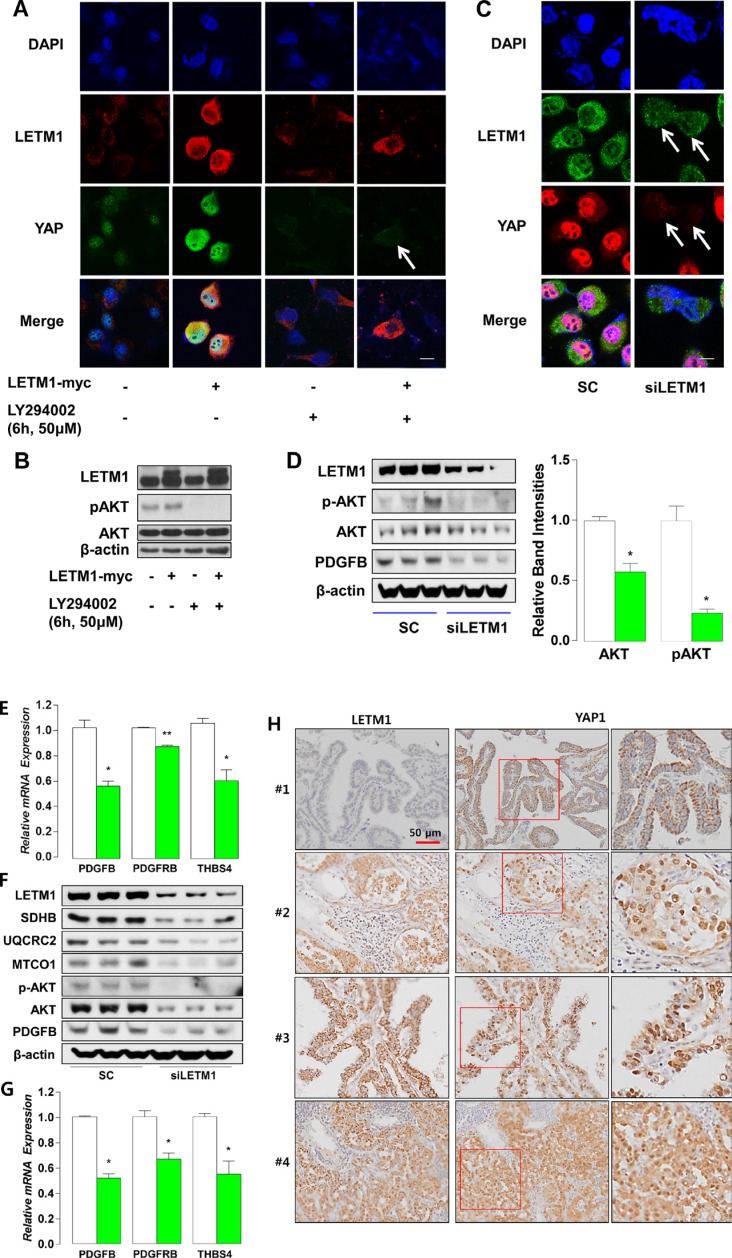
Subcellular localization of YAP1 is modified by LETM1 over-expression or silencing (**A**) Representative immunofluorescence staining indicating increased nuclear YAP1 translocation as a result of LETM1 overexpression, mediated via the PI3K-PKB/AKT signaling pathway in BCPAP cells. White arrow indicates nucleus. (**B**) Representative western blot analyses indicating the effect of LY294002 on AKT phosphorylation. (**C**) Immunofluorescence staining demonstrating the effect of siLETM1 on YAP1 nuclear signal. White arrows indicate nuclei. SC: scramble SiRNA. (**D**) Western blot analyses showing the effect of siLETM1 on total/phospho-PKB/AKT and PDGFB in BCPAP cells. (**E**) qPCR analyses showing the effect of LETM1 silencing on mRNA expression of PDGFB, PDGFRB and THBS4 in BCPAP cells. (**F**) Western blot analyses showing the effect of siLETM1 on total/phospho-PKB/AKT and OxPhos/PDGFB in 8505C cells. (**G**) qPCR analyses showing the effect of LETM1 silencing on mRNA expression of PDGFB, PDGFRB and THBS4 in 8505C cells. Comparisons of the mean values were performed with the Mann-Whitney *U-test*. Data are presented as the means ± SD. ^**^ *P* < 0.01, ^***^ *P* < 0.001. All *P*-values are two-sided. (**H**) Representative results of immunohistochemical staining using anti-LETM1 and YAP1 antibodies (200 x). Case #1, cytosolic localization of YAP1 in PTCs showing no LETM1 staining. Cases #2–4, nuclear accumulation of YAP1 in PTCs showing strong LETM1 staining. Red boxes indicate the areas magnified at the next high power field (far right panel).

YAP1 transactivation is regulated by subcellular localization of YAP1 [22]. When LATS phosphorylates YAP1 at serine 127 residue, YAP1 is exported from nucleus and transactivation is turned off [21]. In contrast, activation of G-protein coupled receptors or receptor tyrosine kinase signaling promotes YAP1 nuclear translocation and YAP1-dependent transcription via PI3K-AKT/PKB signaling activation [23, 24]. Consistent with this regulatory mechanism of YAP1 subcellular localization, when we performed double immunofluorescence (IF) staining for YAP1 and LETM1, we consistently observed that LETM1 overexpression increased the nuclear accumulation of YAP1 in BCPAP cells (Figure 4A). Next, to investigate the involvement of the PI3K-PKB/AKT signaling pathway in YAP1 regulation by LETM1, we treated BCPAP cells with LY294002, a PI3K inhibitor, and performed IF staining. Remarkably, LY294002 was able to abolish the nuclear signal of YAP1 (Figure 4A), which was associated with the disappearance of AKT phosphorylation (Figure 4B). To verify YAP1 regulation by LETM1, we also treated BCPAP cells with siLETM1 and observed a markedly decreased YAP1 nuclear signal (Figure 4C), accompanied by disappearance of AKT phosphorylation (Figure 4D). Taken together, nuclear accumulation of YAP1 could be prevented by the inactivation of PI3K-AKT signaling or down-regulation of LETM1.

### Clinical impact of LETM1 up-regulation in thyroid cancer

Reinforcing our observations regarding the relationship of LETM1 with cellular growth signaling, LETM1 silencing resulted in decreased protein levels of PDGFB and mRNA expression of PDGFB, PDGFBR and THBS4 in BCPAP cells (Figure 4D and 4E) and 8505C cells (human anaplastic thyroid cancer cell line, Figure 4F and 4G). Furthermore, consistent with the IF data, PTC with high immunohistochemical staining (IHC-P) scores of LETM1 showed moderate to strong signals for nuclear YAP1 and PDGFB (Figure 4H, Table 1). To investigate the impact of LETM1 over-expression on clinical outcome, we performed statistical analyses of the relationship between LETM1 IHC staining intensities and clinico-pathological parameters. Remarkably, the lymphovascular invasion (LVI) and lymph node metastasis (LNM) were more frequently observed in patients with PTC showing moderate to strong LETM1 staining intensities (Table 2). In addition, multivariate analysis clearly indicated that moderate to strong LETM1 staining intensities increased risks of LVI and LNM even after adjusting the clinico-pathological parameters (OR = 4.724, CI = 1.423–15.688, Table 3; OR = 2.333, CI = 1.059–5.141, Table 4).

**Table 1:** Protein expression levels of YAP1 and PDGFB according to LETM1 expression status

	Patients with no/focal staining	Patients with moderate strong staining	*P*-value
	*N* = 94 (%)	*N* = 83 (%)	
YAP1 staining			
Focal	19 (20.2)	9 (10.8)	
Moderate	58 (61.7)	55 (66.3)	0.213
Strong	17 (18.1)	19 (22.9)	
YAP1 localization			
Cytosol	17 (18.1)	1 (1.2)	
Nucleus	51 (54.3)	60 (72.3)	0.001
Cytosol+ Nucleus	26 (27.6)	22 (26.5)	
PDGFB staining			
Negative	17 (18.1)	3 (3.6)	
Focal	26 (27.6)	16 (19.3)	0.007
Moderate	22 (23.4)	24 (28.9)	
Strong	29 (30.9)	40 (48.2)	

All *P*-values were calculated from comparisons between three or four groups in linear by linear association.

**Table 2:** Clinicopathologic characteristics according to LETM1 expression status

	Patients with no/focal staining	Patients with moderate to strong staining	*P*-value
	*N* = 94 (%)	*N* = 83 (%)	
Age (years)	42.4 ± 14.8	46.5 ± 14.2	0.111^a^
Gender (Male:Female)	18 (19.1) : 76 (80.9)	13 (15.7) : 70 (84.3)	0.543^b^
Tumor Size (cm)	2.30 ± 1.20	2.23 ± 0.93	0.661^a^
Extrathyroidal Extension			
Negative	31 (33.0)	24 (28.9)	0.560^b^
Positive	63 (67.0)	59 (71.1)	
Multifocality			
Negative	68 (72.3)	64 (77.1)	
Positive	26 (27.7)	19 (22.9)	0.467^b^
Bilaterality			
Negative	77 (81.9)	70 (84.3)	0.668^b^
Positive	17 (18.1)	13 (15.7)	
Lymphovascular invasion			
Negative	72 (76.6)	39 (47.0)	< 0.001^b^
Positive	22 (23.4)	44 (53.0)	
Lymph node metastasis			
Negative	53 (56.4)	31 (37.3)	0.011^b^
Positive	41 (43.6)	52 (62.7)	
Tumor Stage			
T1	17 (18.1)	18 (21.7)	
T2	14 (14.9)	6 (7.2)	0.312^b^
T3	57 (60.6)	56 (67.5)	
T4a/b	6 (6.4)	3 (3.6)	
Lymph Node Stage			
N0	53 (56.4)	31 (37.3)	
N1a	31 (33.0)	39 (47.0)	0.041^b^
N1b	10 (10.6)	13 (15.7)	
Distant Metastasis			
Negative	93 (98.9)	80 (96.4)	0.255^b^
Positive	1 (1.1)	3 (3.6)	
TNM Stage			
I	46 (48.9)	32 (38.6)	
II	2 (2.1)	4 (4.8)	0.658^c^
III	21 (22.3)	19 (22.9)	
IVA	22 (23.4)	26 (31.3)	
IVB	2 (2.1)	1 (1.2)	
IVC	1 (1.2)	1 (1.2)	

a Data are presented as means ± SD and *P*-values were calculated by an independent samples *t*-test.

b *P*-values were calculated by pair-wise comparison using Pearson's χ^2^ test or Fisher's exact test.

c *P*-values were calculated by comparison between three or four groups in linear by linear association.

**Table 3:** Multivariate analysis of the association of lymphovascular invasion (LVI) with LETM1 expression levels

	LVI		
	Odds ratio	95% CI	*P*-value
Moderate to strong staining ^a^	3.690	1.934–7.042	< 0.001
Moderate to strong staining ^b^	3.900	1.886–8.066	< 0.001
Moderate to strong staining ^c^	3.455	1.537–7.766	0.003

a Adjusted for age and sex.

b In addition to adjustment^a^, adjusted for tumor size, multifocality, bilaterality, ETE, LNM, T staging, and M staging.

c In addition to adjustment^b^, adjusted for YAP1 staining, YAP1 localization and PDGFB staining.

Abbreviations: ETE, extrathyroidal extension; LNM, lymph node metastasis; LVI, lymphovascular invasion; CI, confidence interval.

**Table 4:** Multivariate analysis of the association of lymph node metastases (LNM) with LETM1 expression levels

	LNM		
	Odds ratio	95% CI	*P*-value
Moderate to strong staining ^a^	2.177	1.189–3.986	0.012
Moderate to strong staining ^b^	2.388	1.115–5.114	0.025
Moderate to strong staining ^c^	3.043	1.282–7.225	0.012

a Adjusted for age and sex.

b In addition to adjustment^a^, adjusted for tumor size, multifocality, bilaterality, ETE, LVI, T staging, and M staging.

c In addition to adjustment^b^, adjusted for YAP1 staining, YAP1 localization and PDGFB staining.

Abbreviations: ETE, extrathyroidal extension; LNM, lymph node metastasis; LVI, lymphovascular invasion; CI, confidence interval.

## DISCUSSION

LETM1, localized in the inner membrane of mitochondria, is an element of Ca^2+^/H^+^ antiporter and functions in mitochondrial ATP production and biogenesis [15]. Recently, some authors have claimed that high expression levels of LETM1 have correlation with poor prognostic factors in human malignancies [19, 20]. In our study, compatible to previous knowledge, the correlation analysis using GeneNetwork indicated that Letm1 mRNA expression levels had strong positive correlation with mRNA expressions of genes related to OxPhos such as Ndufv1, Sdhb, Cyc1, Atp5b and so forth. Interestingly, our GSEA using repository data by the International Pathology Panel of the Chernobyl Tissue Bank (GSE33630) indicated that PDGF signaling was coordinately enriched in high LETM1 expression PTC. Furthermore, our analyses using TCGA database consistently indicated that PTCs have high expression levels of LETM1 compared to normal thyroid tissue and that high LETM1 expression was significantly associated with expression of genes related to OxPhos. Remarkably, high LETM1 expression was also positively related to glycolysis/gluconeogenesis and cellular growth signaling pathway gene sets. Otto Warburg hypothesized that cancer cell proliferation is facilitated by anaerobic glycolysis known as fermentation. As a result, cancer cell should have dysfunctional mitochondria [25, 26]. However, metabolic heterogeneity may have an impact on tumor behavior and drug responsiveness [11, 13]. Our study implied that the mitochondrial protein LETM1 may enhance ATP generation in cancer cells. Based on the resulting increase in energy supply, cancer cells might be able to generate their own proliferation signal.

The binding of insulin with its ligand specific receptor increases glucose metabolism, lipid synthesis and cellular proliferation via PKB/AKT signaling [27, 28]. In fact, dysregulation of PKB/AKT signaling provokes a broad range of diseases such as cancer, diabetes and heart disease [29, 30]. CTMP was first identified as a PKB/AKT binding partner with tumor-suppressor function. PKB/AKT is negatively regulated by the binding of CTMP with the C-terminal regulatory domain of pPKB/AKT [31, 32]. Together with CTMP, LETM1 is associated with mitochondrial morphology via optic atrophy 1 (OPA1) regulation [33]. And, a recent report demonstrated that LETM1 and CTMP participate in insulin signaling via regulation of PKB/AKT activity [18]. Our GSEA and qRT-PCR results also supported that these LETM1-CTMP-PKB/AKT signaling network might be operational. Taken together, these data suggest that LETM1 might enhance AKT activation. However, this hypothesis must be tested in future studies.

A new finding in this study was the relation of LETM1 with PDGF signaling via YAP1 transactivation. Mammalian Hippo signaling induces inactive YAP1 phosphorylation through MST1/2-LATS1/2 signal propagation [22, 34]. Whereas, activation of receptor tyrosine kinase (RTK) or G-protein coupled receptor (GPCR) turns off Hippo signaling via PI3K activation [23, 24, 35]. Finally, YAP1 moves into nucleus and induces the transcriptions of YAP1 target genes with cell-type specific manner [36]. Based on our volcano plot presenting increased mRNA expressions of PDGFB and CORO2B, representative YAP1 target genes [21], we hypothesized that YAP1 transactivation by PI3K/AKT might be linked to PDGFB up-regulation in high LETM1 PTC, supported by the data from our IF and IHC-P. PDGF is a growth factor promoting cell growth and division especially in blood vessel formation [37]. Up-regulation of PDGF signal in cancer cells results in increased proliferation and cell migration by MEK/ERK activation and lamellipodia/filopodia formation [38, 39]. Compatibly, LETM1 over-expression increased risks of LVI and LNM in our clinical data analyses.

In summary, our bioinformatics and clinical analyses consistently indicated that LETM1 is associated with mitochondrial function and PKB/AKT signaling. Moreover, YAP1 transactivation might have a pivotal role in LETM1-induced PDGF signaling activation. Future study will be focusing on further mechanistic explanations of LETM1-PKB/AKT-YAP1-PDGF signal pathway to develop new druggable targets for invasive or metastatic thyroid cancers.

## MATERIALS AND METHODS

### Subjects and clinical data

A total of 177 patients (31 male and 146 female) who underwent thyroidectomy with central neck node dissection for the management of conventional PTC between March 2014 and December 2014 at Severance Hospital, Yonsei Cancer Center, Seoul, South Korea were enrolled in the study. Samples were taken from the center of the cancer and validated by hematoxylin-eosin staining. On histological examination, > 90% of the cells were confirmed to be thyroid cancer cells. All protocols were approved by the institutional review board of Severance Hospital.

### RNA isolation and real-time PCR

Total RNA was extracted from fresh frozen tissues by Trizol reagent (Invitrogen, Carlsbad, CA, USA), and the RNA quality was verified using a 2100 Bioanalyzer System (Agilent Technologies, Santa Clara, CA, USA). Complementary DNA (cDNA) was prepared from total RNA using M-MLV Reverse Transcriptase (Invitrogen) and oligo-dT primers (Promega, Madison, WI, USA). Quantative RT-PCR (qRT-PCR) was performed using a QuantiTect SYBR^®^ Green RT-PCR Kit (Qiagen, Valencia, CA, USA). Relative expression was calculated using the StepOne™ Real-Time PCR System (Applied Biosystems, Foster City, CA, USA). Primers used in qRT-PCR are listed in Supplementary Table S2. qRT-PCR experiments were repeated three times, and each experiment was performed in triplicate.

### Cell culture and transfection

The human thyroid cancer cell lines BCPAP and 8505C were cultured in RPMI-1640 medium (Hyclone, UT, USA) containing 10% fetal bovine serum (Life Technologies, Carlsbad, CA) and 1% penicillin/streptomycin (Life Technologies). The cells were transfected transiently with pcDNA3.1/LETM1-Myc using jetPEI (Polyplus transfection, Illkirch, France).

### Small interfering RNA (siRNA) transfection and reagents

To knock down endogenous LETM1, cells were transiently transfected with 10 μM chemically synthesized siRNAs (sc-89079, Santa Cruz Biotechnology) targeting LETM1 or with non-silencing control siRNA using Transfection Reagent, according to the manufacturer's recommendations. LY294002 was purchased from Cayman (Ann Arbor, MI, USA). All experiments were performed in duplicate and were repeated at least three times.

### Immunoblot analysis

Immunoblot analysis was performed using standard methods with commercially available antibodies: anti-LETM1 (HPA 011029, Sigma-Aldrich, St. Louis, Missouri, USA), anti- Phospho-AKT (Ser473) (#9271, Cell Signaling, Danvers, MA, USA), anti-AKT (#9272, Cell Signaling), anti-YAP1 (#14074, Cell Signaling), anti-PDGFB (HPA-011972, Sigma), Mitoprofile Total OxPhos Rodent WB Antibody Cocktail (Mitosciences/Abcam, Cambridge, MA, ab110413), anti-HSP60 (#12165, Cell Signaling) and anti-Actin (sc-1616, Santa Cruz Biotechnology, Dallas, TX, USA). The signal intensities on western blots were quantified using ImageJ software.

### Immunofluorescence staining

The cells were plated on coverslips in six-well plates. At 2 days after transient transfection with pcDNA3.1/Myc-LETM1 using jetPEI transfection reagent (Polyplus Transfection, Illkirch, France) (or At 2 days after transfection with siRNA-LETM1 using RNAiMAX transfection reagent (Invitrogen, CA, USA)), the cells were subjected to immunofluorescence staining as described previously [40]. The cells were fixed and permeabilized using conventional methods, and then blocked in 1% bovine serum albumin (BSA). Thereafter, the cells were incubated with anti-YAP1 (#14074, Cell Signaling) and anti-LETM1 (sc-271234, Santa Cruz Biotechnology, Dallas) antibodies for double immunostaining at a 1:100 dilution in 1% BSA for 24 h at 4°C. After washing, cells were incubated with FITC (Fluorescein isothiocyanate)-conjugated pure donkey anti-rabbit and Cy3 (Cyanine)-conjugated pure donkey anti-mouse antibodies (Jackson ImmunoResearch Laboratories Inc., West Grove, PA, USA) at a 1:100 dilution in 1% BSA for 1 h at room temperature in dark. After washing the cells, cells on the coverslips were mounted on glass slides using mounting medium with 4, 6-diamidino-2-phenylindole (DAPI) (Vector Laboratories, Inc., Burlingame, CA, USA) and visualized with a Zeiss LSM710 confocal microscope (Carl Zeiss AG, Jena, Germany). All experiments were performed in duplicate and were repeated at least five times.

### Measurement of oxygen consumption rate and glycolysis

Cells overexpressing or silenced with respect to LETM1 were subjected to mitochondrial oxygen consumption rate (OCR) and extracellular acidification rate (ECAR) measurements using an XF-24 analyzer (Seahorse Bioscience Inc, North Billerica, MA). Three baseline measurements of the OCR and ECAR were made.

### Immunohistochemical analysis

Formalin fixed paraffin-embedded (FFPE) tissue sections (4 μm thick) were deparaffinized and re-hydrated in xylene and a graded series of ethanol, respectively. Antigens were retrieved in 0.01 M citrate buffer (pH 6.0) by microwaving for 10 min. To inactivate endogenous peroxidases, the tissue sections were placed in 3% hydrogen peroxide for 5 min and then blocked for 10 min with normal goat serum. The primary antibodies used for this study were anti-LETM1, anti-PDGFB, and anti-YAP1. Staining scores were assigned as follows: 1, no staining; 2, weak or focal staining; 3, moderate staining in most cells; and 4, strong staining in most cells.

### Public data and statistical analysis

Public repository microarray data from The Cancer Genome Atlas (TCGA, https://tcga-data.nci.nih.gov/tcga/) and the Gene Expression Omnibus (GEO) of NCBI (Gene expression data available at www.ncbi.nlm.nih.gov/projects/geo; accession no. GSE33630) were subjected to GSEA. Data from the GeneNetwork (a free scientific web resource, http://www.genenetwork.org/) were also subjected to analysis. Statistical analysis was carried out using SPSS version 20.0 for Windows (IBM Corporation, Armonk, New York, USA) or GraphPad Prism (GraphPad Software, Inc., San Diego, CA, USA). Data are presented as the means ± SD. All *P*-values are two sided.

## SUPPLEMENTARY MATERIAL TABLES AND FIGURES


