# Supplementary material for: Coupling of LETM1 up-regulation with oxidative phosphorylation and platelet-derived growth factor receptor signaling via YAP1 transactivation
Source: Oncotarget. 2016 Aug 20;7(41):66728–39. doi: 10.18632/oncotarget.11456 (PMC5341833; doi:10.18632/oncotarget.11456)
Supplement: Supplementary file 1 [file oncotarget-07-66728-s001.pdf]

Coupling of LETM1 up-regulation with oxidative phosphorylation and platelet-derived growth factor receptor signaling via YAP1 transactivation

Supplementary Materials

Supplementary Table S1: Top 20 KEGG gene sets enriched in PTCs with highest *LETM1* expression from TCGA

Gene Sets	NES	NOM *p*-val	FDR *q*-val
KEGG_OXIDATIVE_PHOSPHORYLATION	−2.4099	0	0
KEGG_PARKINSONS_DISEASE	−2.34014	0	0
KEGG_VALINE_LEUCINE_AND_ISOLEUCINE_DEGRADATION	−2.23087	0	0
KEGG_BUTANOATE_METABOLISM	−2.22712	0	0
KEGG_LYSINE_DEGRADATION	−2.09001	0	1.39E-04
KEGG_FATTY_ACID_METABOLISM	−2.06724	0	1.16E-04
KEGG_ASCORBATE_AND_ALDARATE_METABOLISM	−1.95948	0	0.001239
KEGG_PROPANOATE_METABOLISM	−1.94719	0	0.001184
KEGG_GLYCINE_SERINE_AND_THREONINE_METABOLISM	−1.92807	0	0.001421
KEGG_BETA_ALANINE_METABOLISM	−1.90651	0	0.002114
KEGG_CITRATE_CYCLE_TCA_CYCLE	−1.8608	0	0.003555
KEGG_HUNTINGTONS_DISEASE	−1.81981	0	0.005337
KEGG_GLYCOLYSIS_GLUCONEOGENESIS	−1.79617	0	0.006428
KEGG_GLYCEROLIPID_METABOLISM	−1.75907	0.001984	0.008891
KEGG_ALZHEIMERS_DISEASE	−1.75154	0	0.009059
KEGG_PPAR_SIGNALING_PATHWAY	−1.71744	0.001859	0.012756
KEGG_PYRUVATE_METABOLISM	−1.6642	0.001942	0.022053
KEGG_CARDIAC_MUSCLE_CONTRACTION	−1.64206	0.003883	0.025365
KEGG_VASCULAR_SMOOTH_MUSCLE_CONTRACTION	−1.56894	0	0.046522
KEGG_PRIMARY_BILE_ACID_BIOSYNTHESIS	−1.52158	0.029358	0.065884
KEGG_TERPENOID_BACKBONE_BIOSYNTHESIS	−1.50885	0.041746	0.069799
KEGG_ARGININE_AND_PROLINE_METABOLISM	−1.47019	0.035417	0.092299
KEGG_PORPHYRIN_AND_CHLOROPHYLL_METABOLISM	−1.46835	0.028	0.08941
KEGG_PEROXISOME	−1.46194	0.016427	0.090055
KEGG_STARCH_AND_SUCROSE_METABOLISM	−1.44732	0.029106	0.096405
KEGG_RETINOL_METABOLISM	−1.41754	0.033465	0.116446
KEGG_DRUG_METABOLISM_CYTOCHROME_P450	−1.41176	0.023715	0.116809
KEGG_INSULIN_SIGNALING_PATHWAY	−1.41066	0.014315	0.113356
KEGG_TRYPTOPHAN_METABOLISM	−1.37986	0.046422	0.136344

NES, Normalized enrichment score.

FDR, False discovery rate.

Supplementary Table S2: List of primer sets used for qRT-PCR

Gene	Forward Primer	Reverse Primer
PDGFB	CTCGATCCGCTCCTTTGATGA	CGTTGGTGCGGTCTATGAG
PDGFRB	AGCACCTTCGTTCTGACCTG	TATTCTCCCGTGTCTAGCCCA
THBS4	TGCTGCCAGTCCTGACAGA	GTTTAAGCGTCCCATCACAGTA
YAP1	TAGCCCTGCGTAGCCAGTTA	TCATGCTTAGTCCACTGTCTGT
CORO2B	CGTCCGCAATACCGTAGCTC	TAGTTGGGTTCAATCCTGCCT
LETM1	CCGAGTGCCTTCGCATAGTG	ACTTCTCTACTACCGAGTCATC

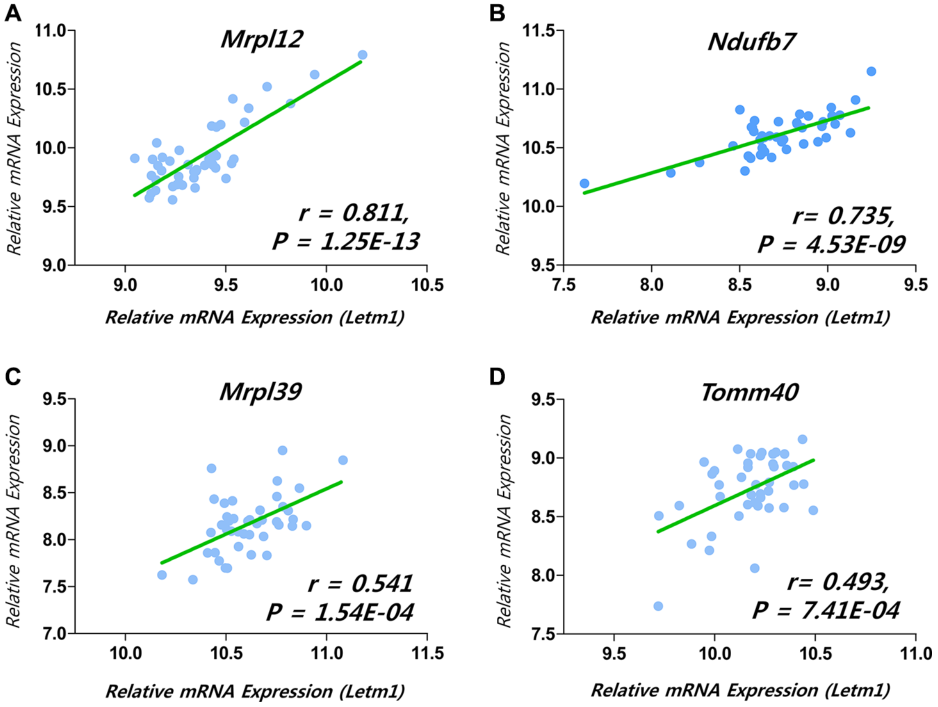

Supplementary Figure S1: (**A**–**D**) Correlation of Letm1 mRNA expression with genes related to mitochondrial ribosomal proteins (Mrpl12 and Mrpl39), Ndufb7 and Tomm40 from public repository data in GeneNetwork (a free scientific web resource, http://www.genenetwork.org/; INIA Adrenal Affy MoGene 1.0ST (Jun12) RMA Males, EPFL/LISP BXD CD Muscle Affy Mouse Gene 1.0 ST (Nov12) RMA Exon Level).

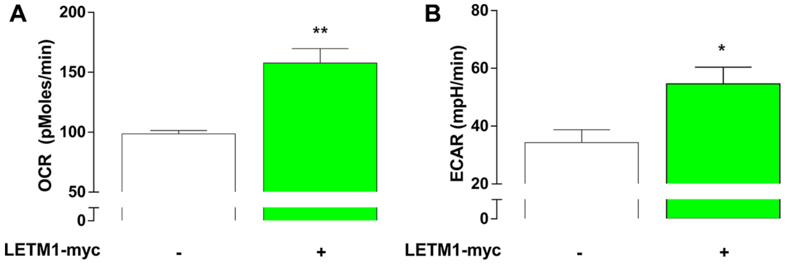

Supplementary Figure S2: (**A**–**B**) Oxygen consumption rate (OCR) and extracellular acidification rate (ECAR) following LETM1 overexpression in BCPAP cells. OCR and ECAR were measured in triplicate under basal conditions. Comparisons between means were performed using two-sided Mann-Whitney *U*-tests. Data are presented as mean ± SD. $^{**}P < 0.01$, $^{***}P < 0.001$.
